# Construction of a heat-resistant strain of *Lentinus edodes* by fungal Hsp20 protein overexpression and genetic transformation

**DOI:** 10.3389/fmicb.2022.1009885

**Published:** 2022-11-17

**Authors:** Yun-Yan Ling, Zhi-Lin Ling, Rui-Lin Zhao

**Affiliations:** ^1^State Key Laboratory of Mycology, Institute of Microbiology, Chinese Academy of Sciences, Beijing, China; ^2^College of Life Science, University of Chinese Academy of Sciences, Beijing, China

**Keywords:** heat shock protein, thermotolerance, genetic transformation, *shiitake*, *HSP*20

## Abstract

The shiitake mushroom (*Lentinus edodes*) is the second most popular edible mushroom globally due to its rich nutritional value and health benefits associated with consumption. However, the characteristics of growing at low temperatures limit the area and time of its cultivating. We selected a low-temperature cultivar as the original strain. We proposed to construct a heat-shock protein expression vector to achieve genetic transformation in this low-temperature strain to improve the survivability of the strain against the heat-shock response. In this study, an overexpression vector pEHg-gdp-*hsp20* for the heat shock protein 20 gene of *A. bisporus* was constructed using a homologous recombination method. This vector was transferred into dikaryotic and monokaryotic mycelia by the *Agrobacterium tumefaciens*-method. The integration of *hygb* and *hsp20* into the genome of *L. edodes* mycelia was verified by growth experiments on resistant plates and PCR analysis. The expression of the reporter gene mgfp5 was verified by fluorescence microscopy analysis and statistically resulted in 18.52 and 26.39% positivity for dikaryon, and monokaryon, respectively. Real-time PCR analysis showed that the expression of the *hsp20* gene was more than 10-fold up-regulated in the three transformants; the mycelia of the three overexpression transformants could resume growth after 24 h heat treatment at 40°C, but the mycelia of the starting strain L087 could not recover growth at 25°C indicating that strains that successfully expressed *hsp20* had greater overall recovery after heat shock. According to the study, *A. bisporus hsp20* gene overexpression effectively improves the defensive capability of low-temperature mushroom strains against heat shock, laying the foundation for breeding heat-resistant high-quality transgenic shiitake mushrooms.

## Introduction

*Lentinula edodes* (Berk.) Pegler is one of the most valuable edible mushrooms in the world. The consumption of *L. edodes* has increased substantially in recent years, with markets expanding from southeast Asia to worldwide. It has been reported that the production of *L. edodes* is approximately 22% of the total mushroom supply all over the world (Royse et al., 2017). The temperature is one of the important environmental factors affecting the growth and development of *L. edodes*. In general, *L. edodes* strains are low-medium temperatures strains, and their optimal temperature range is 24–27°C (Zhao et al., 2016). The hot summer weather can adversely affect the quality and yield of the mushroom (Cao et al., 2015), thereby inhibiting the development of the industry. High-temperature environments can lead to a decrease in the viability of mycelia (Zhang et al., 2016). The mycelial growth rate at 30°C is only half or one-third of that at 25°C. High temperatures also cause *Trichoderma* to infest shiitake mycelia, which is referred to as the “fungal burning phenomenon “(Guo et al., 2020). Furthermore, the agronomic traits of fruiting body are also affected, as evidenced by the smaller mushrooms, loose texture, thin pileus, and easily opened caps (Zhao et al., 2018). Finally, mycelial death will occur at temperatures exceeding a specific threshold (above 38°C; Wang et al., 2004).

Heat stress has become one of the most important abiotic stresses for crops (Hasanuzzaman et al., 2013). However, organisms respond to heat stress in a variety of ways, such as heat shock family proteins (HSP), phytohormones, redox proteins and signal transduction pathway proteins (e.g., Ca^2+^). These expressions are positively correlated with heat tolerance in organisms at the molecular level and metabolite networks (Kotak et al., 2007; Martín-Folgar et al., 2015). HSPs are conserved proteins that accumulate in large quantities at high temperatures and play a crucial role in protein renaturation, enzyme and membrane stability, and cell homeostasis (Morano et al., 2012; Park and Seo, 2015). *HSP100*, *HSP90*, and *HSP70* can refold and activate misfolded proteins, preventing misfolded protein aggregation, improving plant and fungal defenses against heat shock responses (Bui et al., 2016). Small heat shock proteins such as *HSP*20 and *HSP*40 can bind to unfolded protein substrates under responding conditions, preventing their irreversible aggregation (Deng et al., 2014). Currently, few studies have been conducted on the mechanism of heat shock response in *L. edodes*. RT-PCR analysis after high-temperature stress showed that the expression of *Hsp40*, *Hsp60*, *Hsp70*, *Hsp90* and *Hspl00* was higher in heat-tolerant strains than in heat-sensitive strains of *L. edodes* (Xin et al., 2016; Wang et al., 2018a). Some domestic studies have shown that the addition of exogenous auxins can improve *L. edodes* mycelial thermotolerance (Zhou et al., 2018); and have studied knockdown and overexpression of the anthranilate-synthase gene (TrpE) in *L. edodes* to investigated its role during heat stress (Ma et al., 2018; Wang et al., 2020).

*Agrobacterium tumefaciens* is a Gram-negative soil bacterium that can deliver a specific segment of its genome to the nucleus of susceptible plant cells by infecting them (Su et al., 2012). De Groot et al. (1998) first used *A. tumefaciens* to transform fungal mycelia. Since then, studies have continued to show that *A. tumefaciens-*mediated transfer (ATMT) enables the random transfer of T-DNA into the genomes of fungi, including ascomycetes, basidiomycete and zygomycete (Poyedinok and Blume, 2018). In recent years, *Cordyceps militaris* (Zheng et al., 2011), *Agaricus bisporus* (Wang et al., 2019), *Flammulina velutipes* (Lyu et al., 2021) and other species have been successfully tested for *A. tumefaciens*-mediated transfer. However, this method has the disadvantage that it is not yet stable and consistent. The advantage is that many transformants can be obtained without the use of special equipment and also increases the frequency of homologous recombination, effectively knocking out the target gene.

The ability of edible fungi to withstand heat stress is a crucial trait that influences their growth and development. Based on heat stress studies in *A. bisporus* (Deng et al., 2014), the *hsp20* gene, an up-regulated expression gene induced after heat stress, was transformed into *L. edodes* mycelia by *Agrobacterium*-mediated method. In this study, we will explore the effect of *hsp20* on mycelial growth and resistance to heat stress in *L. edodes*, intending to improve the defense ability of low-temperature strains against heat shock response and lay the groundwork for breeding heat-tolerant strains.

## Materials and methods

### Strains and culture conditions

We used the monokaryotic LeL10-SSI17 derived from the strain 79,016 (source from the China Agricultural Microbial Strain Conservation Centre, the number is 5.0174) and the dikaryotic strain L087 of *L. edodes* for genetic transformation. These strains mentioned above are conserved and provided by our group. Strains and transformants were maintained on potato dextrose agar medium (PDA) at 25°C. *E. coli* strain DH5α and *A. tumefaciens* strain EHA105 were cultured in Luria-Bertani media (LB) at 28°C.

### Nucleic acid extraction and PCR amplification

We extracted genomic DNA from *L. edodes* mycelia using the CTAB method. The promoter of the *L. edodes* glyceraldehyde-3-phosphate dehydrogenase gene (Legpd), was amplified from *L. edodes* DNA by primers Legpd-F/Legpd-R. The PCR product was purified and sequenced by the BGI Genomics. Mycelia from *A. bisporus* were cultured in liquid PDA medium at 24°C, 160 r/min for 2 weeks, then exposed to sublethal temperature (30°C) for 3 h of heat induction, followed by 12 h of heat treatment at lethal temperature (37°C; Lu, 2010). By rapid filtration, mycelia were ground in liquid argon and total RNA was extracted using Trizol method. RNA was reverse transcribed to cDNA (42°C at 15 min and 95°C at 3 min) using FastKing gDNA Dispelling RT SuperMix kit (Tiangen, China). The *hsp*20 gene was amplified from *A. bisporus* cDNA by primers hsp20-F /hsp20-R. The primers were synthesized by BGI Genomics, and the sequences are listed in Appendix 1.

### Plasmid construction

**Figure 1:**
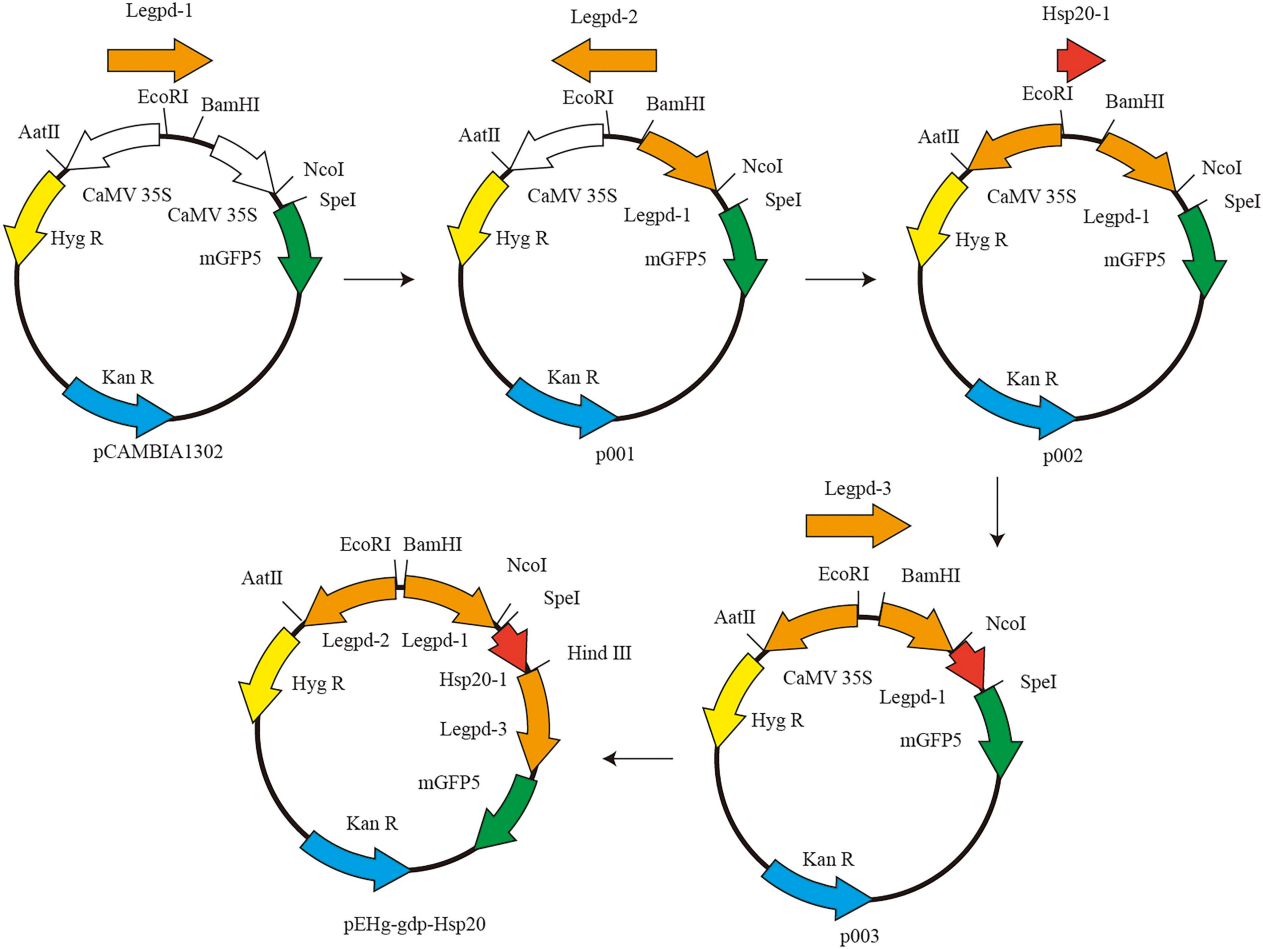
The schematic diagram of plasmid pEHg-gdp-*hsp20.*

The vector plasmid pCAMBIA1302 was purchased from Beijing Dingguo Changsheng Biotechnology Co. pCAMBIA1302 contains two genes, hygromycin resistant (*hygR*) gene and green fluorescent protein (*mgfp5*) gene, both of which are activated by the cauliflower mosaic virus (CaMV) 35S promoter. The CaMV 35S promoter cannot be used for genetic transformation in *L. edodes* and must be replaced with a corresponding homologous promoter The schematic diagram of plasmid construction is shown in Figure 1. First, pCAMBIA1302 was double digested with restriction endonucleases *BamH*I and *Nco*I (New England Biolabs, USA). Legpd-1 was amplified using *L. edodes* DNA as template and Legpd-F1/Legpd-R1 as primers. Linearized pCAMBIA1302 was ligated with Legpd-1 to produce the recombinant plasmid p001. Second, p001 was double digested with *EcoR*I and *Aat*II (New England Biolabs, USA). Reverse Legpd-2 (synthesized by Beijing Genomics institution) ligated with linearized plasmid p001 to produce the intermediate plasmid p002. Third, p002 was single digested with *Spe*I (New England Biolabs, USA). Linearized plasmid p002 was ligated with hsp20-1 with a homologous sequence junction and Hind III cleavage site to produce the intermediate plasmid p003. Finally, p003 was single digested with *Hind*III (New England Biolabs, USA). Legpd-3 was amplified using *L. edodes* DNA as template and Legpd-F3/Legpd-R3 as primers. Linearized p003 was ligated with Legpd-3 to produce the recombinant plasmid pEHg-gdp-*hsp20*. This recombinant plasmid contains the Legpd-1 promoter expresses the *hsp20* protein gene, the Legpd-2 promoter expresses the hyg resistance gene, and the Legpd-3 promoter expresses the mGFP5 reporter gene.

### Hygromycin and cefotaxime sodium susceptibility assays

We conducted experiments to determine the minimum hygromycin concentration that kills *L. edodes* mycelia. Agar discs with *L. edodes* mycelia (*d = 8 mm*) were cut with a sterile drill from the edge of the actively growing colonies and transferred to solid MYG media containing hygromycin B (Hyg B) at concentrations of 0, 6, 8, 10, 12, 14, 16 and 18 μg/ml at 25°C for 10 days. The three biological replicates were set up for each group. Starting on the fifth day, colony diameters were measured daily to determine the susceptibility of *L. edodes* mycelia to Hyg B.

For genetic transformation, *A. tumefaciens* and *L. edodes* must co-cultivate, during which a large amount of *A. tumefaciens* is invariably mixed with *L. edodes* mycelia. *A. tumefaciens* overgrowth inhibits the growth of *L. edodes* mycelia (Chen et al., 2009), therefore Cefotaxime sodium (Cef) is necessary to eradicate *A. tumefaciens*. We conducted experiments to determine the effect of Cef concentration on mycelial growth. Agar discs with *L. edodes* mycelia (*d = 8 mm*) were cut with a sterile drill from the edge of the actively growing colonies and transferred to solid MYG medium containing Cef at concentrations of 0, 200, 400, 600 μg/ml at 25°C for 10 days. The three biological replicates were set up for each group. Starting on the fifth day, colony diameters were measured daily to determine the susceptibility of *L. edodes* mycelia to Cef.

### *Agrobacterium tumefaciens*-mediated transformation

The activated *L. edodes* strains were transferred to solid MYG media and incubated at 25°C until the colonies were 5.5–6 cm in diameter. The selected vector pEHg-gdp-*hsp20* was introduced into *A. tumefaciens* EHA105 using electroporation. The transformed EHA105 were grown in 100 ml LB liquid media containing 50 μg/ml Kanamycin (Kan), incubated at 28°C with 180 rpm until OD_600_ reached about 0.5–0.8. The bacterial suspension was collected in a sterile 50 ml centrifuge tube and centrifuged at 4500 rpm for 12 min. After centrifugation, the supernatant was carefully removed and 30 ml of liquid induction medium (IM) containing 200 μM AS and 40 mM MES was added to resuspend the bacteria. We attempted to mix *A. tumefaciens* sufficiently with the liquid IM medium to induce its transformation. Agar discs with *L. edodes* mycelia (*d* = 8 mm) were cut with a sterile drill from the edge of the actively growing colonies and transferred to *A. tumefaciens-*IM mix until the centrifuge tube was filled. The mixture of mycelia and *A. tumefaciens-*IM were incubated at 25°C for 4–6 h. The supernatant was carefully removed, the surface of agar discs was blotted out with sterile filter paper, and the discs were placed in co-cultivation IM media (containing 200 μM AS and 40 mM MES), covered with cellophane at 25°C for 3–5 days. In this period, white mycelia were observed sprouting on the surface of the agar discs. After co-culture, agar discs were washed with sterile water containing Cef, blotted dry with sterile filter paper and transferred to solid MYG media (containing 12 μg/ml Hyg B and 400 μg/ml Cef). Nine agar discs were placed on each plate and incubated at 25°C until transformants grew. Many mycelia grew at the early stages of the culture, but after 6–7 days, most of them ceased to grow except for the proposed transformants. After the first screening, the single colonies were transferred to a new MYG media (containing 12 μg/ml Hyg B and 400 μg/ml Cef) and screened for five generations. All the steps mentioned above were carried out on a clean bench.

### Molecular analysis and fluorescence observation

The first selection of well-grown single colonies was transferred to MYG media covered with cellophane at 25°C until mycelia were fully grown. We extracted genomic DNA from mycelia using the CTAB method. The presence of *hyg* and *hsp20* genes was demonstrated by polymerase chain reaction (PCR) analysis using two primer pairs: *hyg*-F1/*hyg*-R1 and *hsp20*-F2/*hsp20*-R2 (Appendix 1). To detect the fluorescent signal of the reporter protein mGFP5, transformants were cultivated in MYG media and coverslips were placed diagonally on the edge of the medium until the mycelia climbed onto the coverslip. The coverslip was then removed and the expression of green fluorescent protein in the mycelia was observed using an upright fluorescence microscope (Zeiss, Germany).

### Gene expression analysis

Positive transformants were inoculated in MYG media covered with cellophane at 25°C until fully grown. We extracted total RNA from mycelia using the SV Total RNA Isolation System Kit (Promega, China) according to protocol of manufacturer. RNA was reverse transcribed to cDNA using the FastKing gDNA Dispelling RT SuperMix kit (Tiangen, China). The reverse transcriptase reaction consisted of 4 μl 5 × FastKing-RT SuperMix, 1 μl total RNA (2 μg/μL) and RNase-free ddH_2_O up to 20 μl. The RT-PCR program was 42°C for 15 min and 95°C for 3 min. The cDNA products were stored at −20°C.

Real-time quantitative PCR (qPCR) was used to detect the expression of the *hsp20* gene in the transformants. The GAPDH (Glyceraldehyde-3-Phosphoglyceraldehyde dehydrogenase) gene (Genbank accession number is AB012862.1) was selected as the internal reference gene for PCR quantification. The primers for the *hsp20* and GAPDH genes were designed and tested for specificity using NCBI’s online program.^1^ The primers were synthesized by the Beijing Genomics institution, and they are listed in Appendix 1. Real-time quantitative PCR was performed with the SuperReal PreMix Plus (SYBR Green) Kit (Tiangen, China). The reaction solution was prepared on ice, and the components were 10 μl 2 × SuperReal PreMix Plus (SYBR Green), 1 μl forward Primer, 1 μl Reverse Primer, 2 μl cDNA, and ddH_2_O up to 20 μl. The qPCR reactions were performed as follows: 94°C for 15 min, followed by 35 cycles at 95°C for 10 s, 60°C for 20 s and 72°C for 30 s. All reactions were run in triplicate. The primers qgapdh-F/R and qhsp20-F/R were used to make standard curves for amplification of gene GAPDH and *hsp20*, respectively. When the primer efficiency (E) was between 90 and 110%, the correlation coefficient (*R*^2^) was determined to be greater than 0.98 and the difference of the amplification efficiency between the target gene and the internal reference gene was within 10%, the relative expression quantification was calculated using the 2^−△△ct^ method after the threshold cycle (*Ct*) and was normalized with the *Ct* of GAPDH.

### Observation of colony morphology and growth rate determination

The untransformed strain L087 and overexpressed transformants were inoculated in fresh PDA media at 25°C for 10 days. Agar discs with mycelia (*d* = 8 mm) were cut with a sterile drill from the edge of the actively growing colonies, and transferred to PDA media at 25°C, with three biological replicates set up for each group. When the mycelia started to sprout, the diameter of the mycelia was measured as r; the diameter of the colony was measured daily as R; when the mycelia had grown to the point of nearly covering the PDA plate, the diameter of the colony was again measured as R. The number of days the colony had grown at this point was recorded as n. The mycelial growth rate was calculated separately according to the formula: *v* = (*R*−*r*)/*n*. Statistical analysis (one-way ANOVA) was performed using GraphPad Prism 8.0 software (*α* = 0.05).

### Thermotolerance susceptibility assays and gene expression analysis for transformants

The untransformed L087 and overexpressed transformants were inoculated in fresh PDA media at 25°C for 5 days, and three biological replicates were set up for each group. Before heat treatment, the mycelial diameter was measured as R. After being heat-treated at 40°C for 24 h, mycelia were incubated at 25°C for 10 days to observe whether they had recovered growth and measure mycelial diameter as r. In order to compare the growth of mycelia before and after heat treatment, the difference in mycelial diameter was calculated. Meanwhile, the expression analysis of the *hsp20* gene in mycelia was examined by real-time PCR.

## Results

### Monokaryon have higher resistance to hygromycin

**Figure 2:**
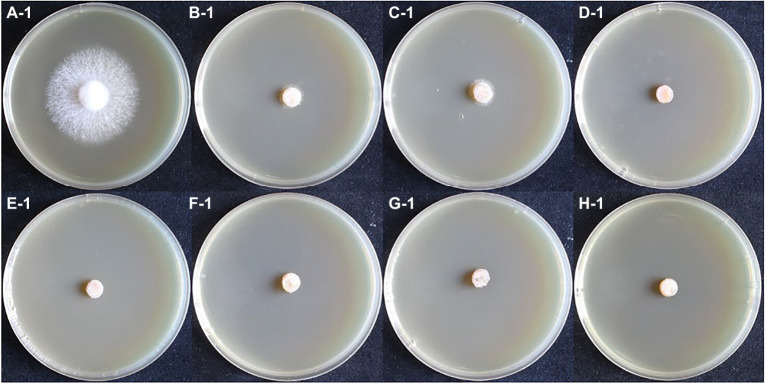
The growth of dikaryotic strain in different hygromycin concentrations. **(A–H)** The concentrations of hygromycin were 0, 6, 8, 10, 12, 14, 16, 18 μg/ml, respectively.

**Figure 3:**
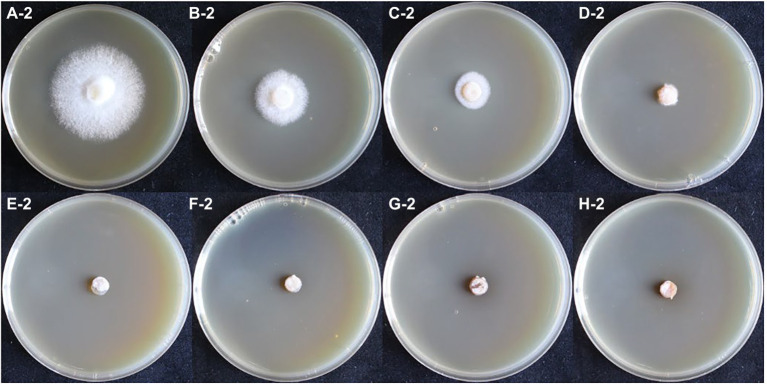
The growth of monokaryotic strain in different hygromycin concentrations. **(A–H)** The concentrations of hygromycin were 0, 6, 8, 10, 12, 14, 16, 18 μg/ml, respectively.

In the hygromycin susceptibility experiments, as shown in Figures 2, 3, mycelial growth was significantly inhibited in all experimental groups compared to the control group. For the growth of the dikaryotic strain (Figure 2): when the hygromycin concentration was between 6 μg/ml and 8 μg/ml, the mycelia could germinate, but could not extend; when the hygromycin concentration exceeded 12 μg/ml, the mycelia were unable to germinate or extend. For the growth of the monokaryotic strain (Figure 3): when the hygromycin concentration was 6 μg/ml and 8 μg/ml, the mycelia could extend but growth was significantly inhibited; when the hygromycin concentrations was 10 μg/ml, the mycelia could germinate, but could not extend; when the hygromycin concentrations was 12 μg/ml, the mycelia were unable to germinate and the color of the agar discs was significantly darkened. The results showed that hygromycin had a significant inhibitory effect on *L. edodes* mycelia and the monokaryotic strain was more resistant to hygromycin than the dikaryotic strain. The concentration of hygromycin in the primary screened MYG medium was set at 12 μg/ml.

### No significant effect of cefotaxime on *Lentinus edodes* growth

**Figure 4:**
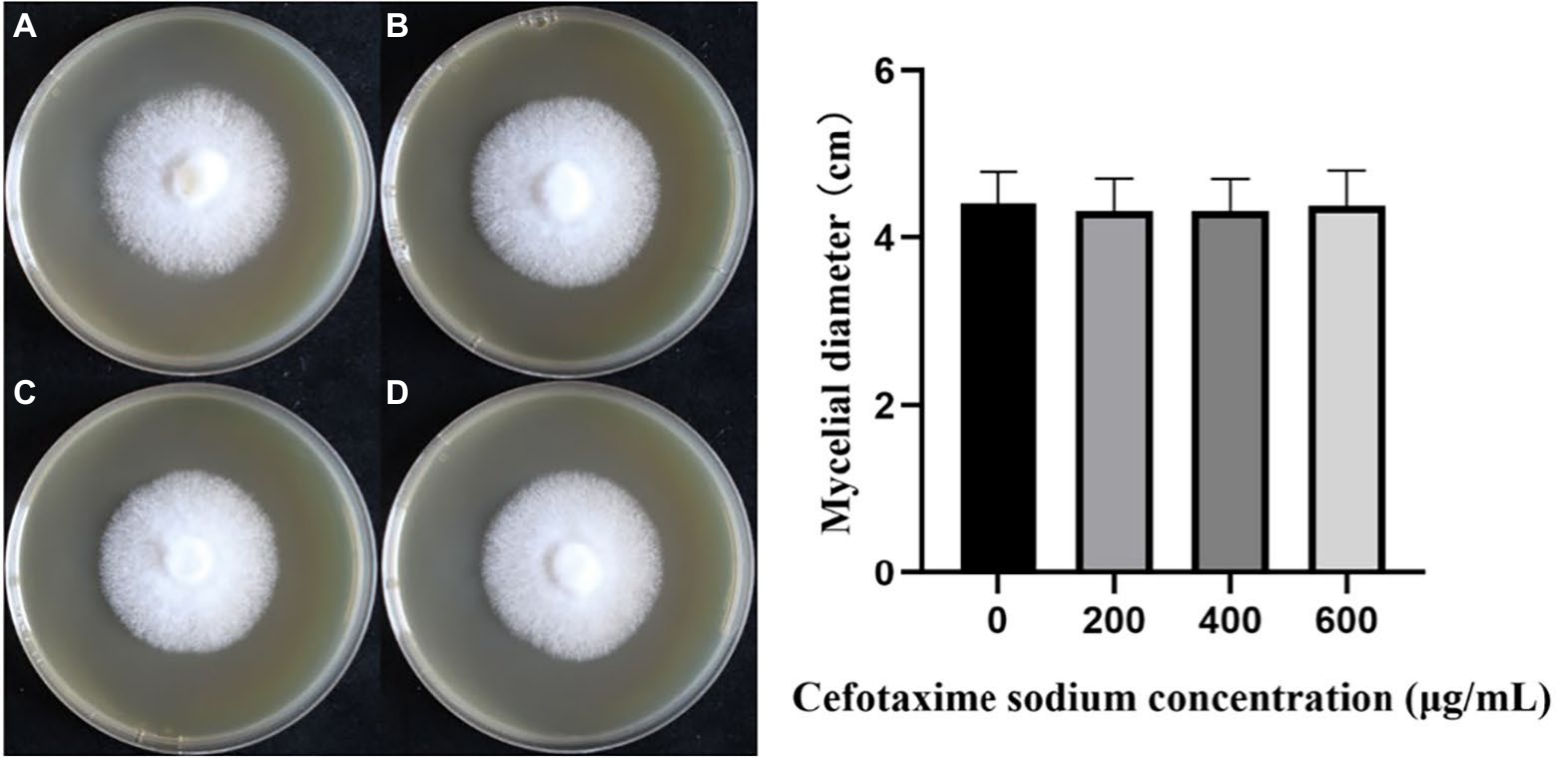
The growth of monokaryotic strain in different cefotaxime concentrations. **(A–D)** The concentrations of cefotaxime were 0, 200, 400, 600 μg/ml, respectively.

In the cefotaxime susceptibility experiments, as shown in Figure 4, when the concentration of Cef in MYG media were 0, 200, 400 and 600 μg/ml, the mycelia of *L. edodes* grew normally without significant differences from the control group. Colony diameters were measured daily and one-way ANOVA (α = 0.05) as shown in Figure 4. There was no significant difference in the growth rate of mycelia with different Cef concentrations. Previous studies on *A. tumefaciens* have demonstrated that 400 μg/ml of Cef can completely inhibit the growth of *A. tumefaciens* with OD_600_ = 0.5 (Wang et al., 2006). The concentration of Cef in the primary screened MYG medium was set at 400 μg/ml.

### HSP20 of *Agaricus bisporus* is conserved protein

The software predicts that HSP20 protein contains 155 amino acids, of which 36–154 are the conserved structural domains, that belong to the IbpA superfamily. The molecular weight of the HSP20 protein is 17.49 kDa and the isoelectric point is 5.74. The HSP20 protein is rich in α-helices (28, 18.06%), extended chains (36, 23.23%), β-folds (10, 6.45%) and randomly coiled structures (81 or 52.26%). Neither a transmembrane structural domain nor a signal peptide is present in the HSP20 protein.

**Figure 5:**
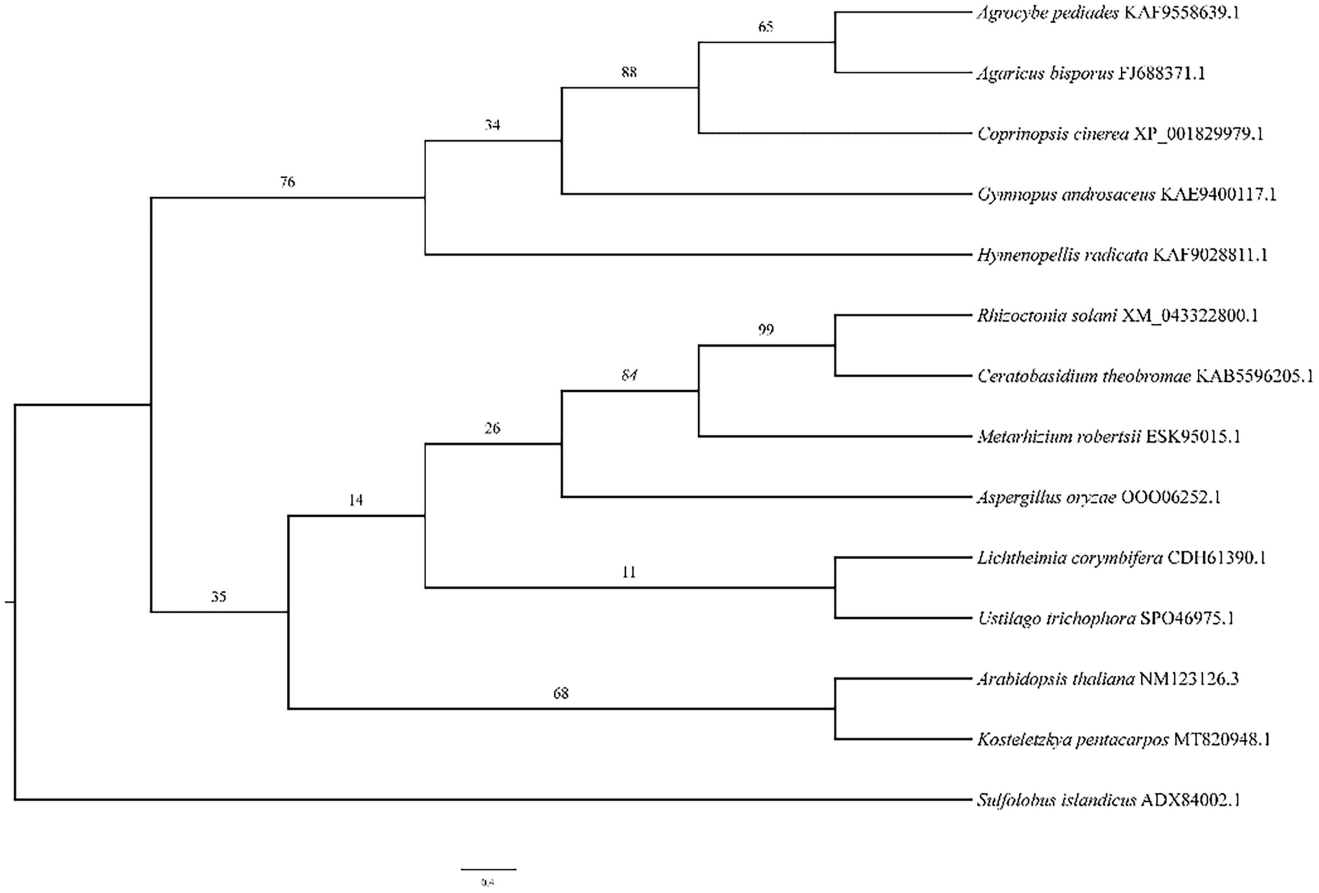
The phylogenetic tree analysis of HSP20 in *A. bisporus.*

The phylogenetic tree (Figure 5) illustrates the relationship between *A. bisporus* and other species, classified into the basidiomycota, zygomycota, deuteromycota and plants. The HSP20 gene of *A. bisporus* belongs to the basidiomycota branch and is more closely related to *Agrocybe pediades* and *Coprinopsis cinerea*, with 76.4 and 61.7% sequence identity respectively; and more distantly related to *Arabidopsis thaliana*, with only 36.7% sequence identity.

### Monokaryon have higher transformation efficiency

**Figure 6:**
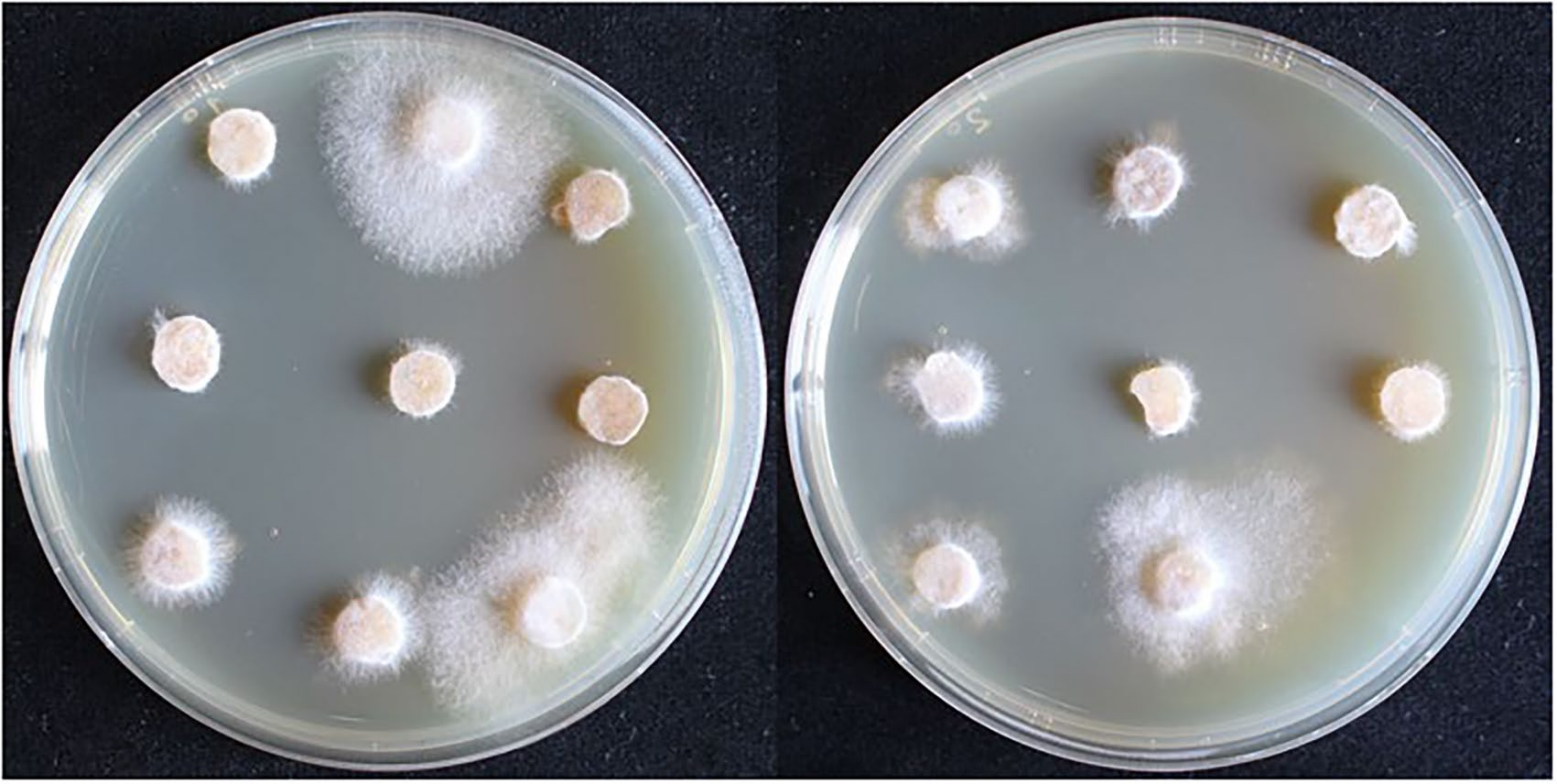
The first selection of dikaryotic transformants in *L. edodes*. The mycelia can germinate and extend on the resistant medium are transformants.

The recombinant plasmid pCAMBIA1302-gpd-*hsp20* was transformed into the dikaryotic strain L087 and the growth of transformants on resistant plates is shown in Figure 6. In the first batch, 117 *A. tumefaciens*-infested mycelia were initially screened and 18 proposed transformants germinated, with a germination rate of 15.38%. In the second batch, 135 infested mycelia were initially screened and 26 proposed transformants were successfully cultivated, with a germination rate of 15.56%. Both replicate experiments demonstrated similar germination rates.

The plasmid pCAMBIA1302-gpd-*hsp20* was transformed into the monokaryotic strain LeL10-SSI17. In the first batch, 72 infested mycelia were initially screened and 19 proposed transformants germinated, with a germination rate of 26.39%. In the second batch, 117 infested mycelia were initially screened and 23 proposed transformants were successfully cultivated, with a germination rate of 19.66%. As a result of our experiments, we can conclude that monokaryon as the acceptor material had a higher transformation efficiency than dikaryon.

### Integration of marker and *hsp20* genes into mycelia

**Figure 7:**
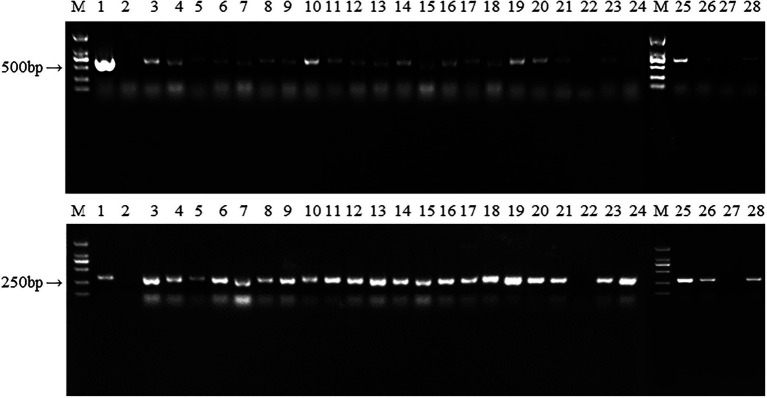
The PCR identification of transformants. M: DL 2000; 1: Plasmid as positive control; 2: untransformed strain L087 as negative control; 3–24: Transformants; M: DL 2000; 25–28: Transformants.

We obtained plenty of hygromycin-resistant transformants after passaging each transformant five times. 26 transformants were randomly selected for PCR analysis to determine the presence of *hygR* and *hsp20*. As shown in Figure 7, 24 transformants and the positive control amplified the *hygR* and *hsp20* bands, with bands of 648 and 368 bp respectively; while untransformed strain L087 and the transformants numbered 22, 27 failed to amplify the bands, indicating that the transformants numbered 22 and 27 were false positives. Thus, we can tentatively conclude that these 24 transformants with bands of expected sizes are positive; the foreign genes *hygR* and *hsp20* were successfully integrated into the genome of *L. edodes*.

### mGFP5 expression in mycelia

**Figure 8:**
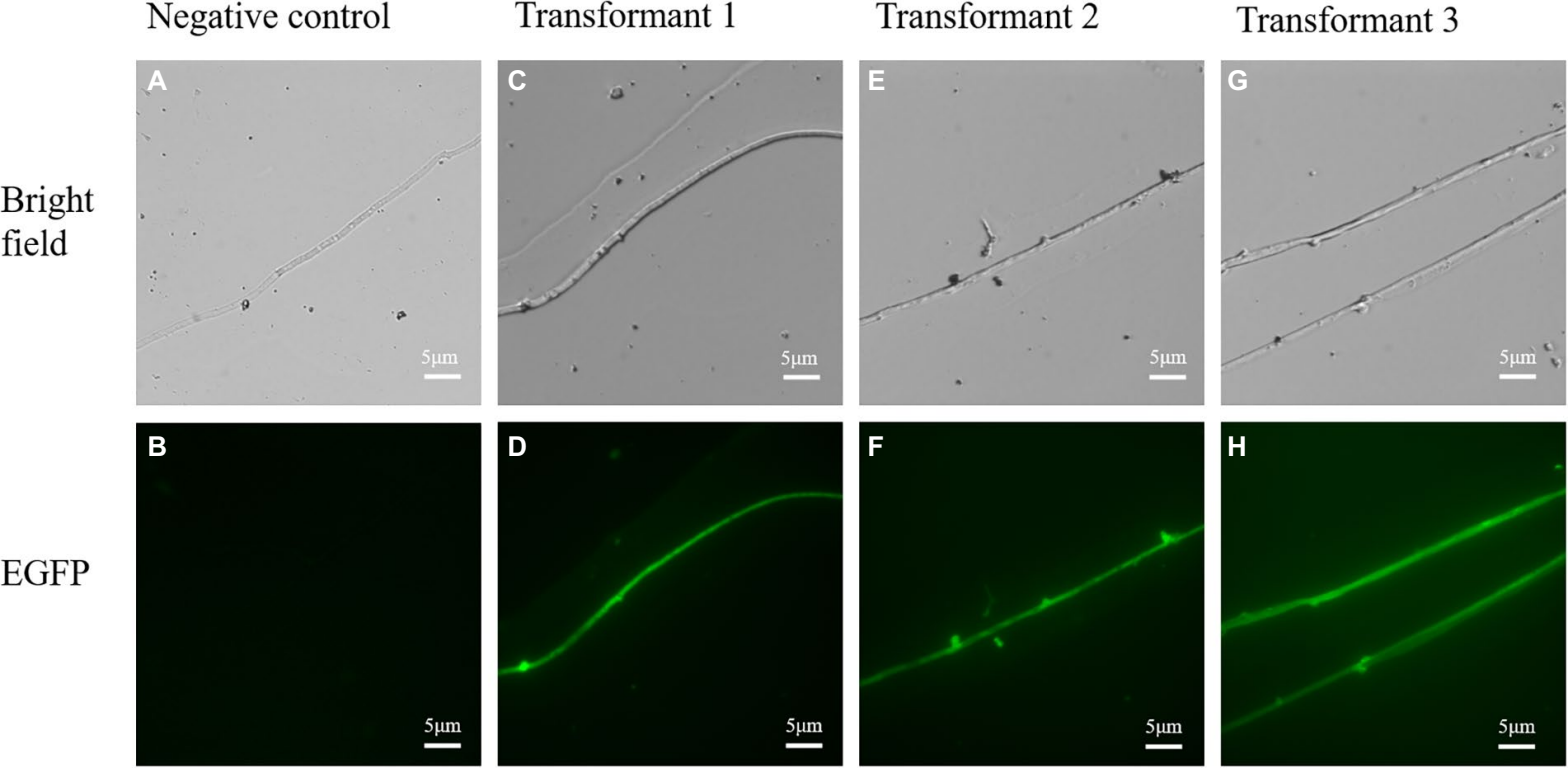
The confocal microscopic assays of randomly selected transformants in *L. edodes*. **(A,B)** Bright field and green fluorescence images of untransformed mycelia. **(C–H)** Bright field and green fluorescence images of randomly chosen transformed mycelia.

To confirm the expression of the mGFP5 reporter gene, the mycelia of transformants and wild strains were examined under an upright fluorescence microscope (Figure 8). The majority of transformants showed a positive green fluorescent signal while there was no fluorescent signal found in the wild-type mycelia. The results indicated that the mGFP5 gene had been integrated into the *L. edodes* genome and expressed, as the positive transformants emitted a strong fluorescence signal when exposed to excitation light.

### Monokaryon have higher positive rate

A total of 86 transformants were obtained from the first screen. The potential transformants were passaged more than five times to obtain stably expressed transformants. These transformants were identified by PCR and fluorescence microscopy observations, and the statistical results are shown in Appendix 2. Some of the potential transformants lost their resistance during the passaging process. When the mycelia failed to grow on resistant plates; a few DNAs could not be identified by PCR to obtain the target gene bands, which we called false positives. We eventually obtained a total of 81 stably expressed transformants.

The plasmid was transformed into the heterokaryotic strain L087. In the first batch, we screened 117 infested mycelia and counted 18 potential transformants. After passaging each transformant five times, PCR identification and fluorescence microscopy, 17 transformants were verified as positive, with a positive rate of 14.52%. In the second batch, we screened 135 infested mycelia and counted 26 potential transformants. Finally, 25 transformants were verified as positive, with a positive rate of 18.52%.

The plasmids were transformed into the monokaryotic strain LeL10-SSI17. In the first batch, we screened 72 infested mycelia and counted 19 potential transformants. After passaging each transformant five times, PCR identification and fluorescence microscopy, 19 transformants were verified as positive, with a positive rate of 26.39%. In the second batch, we screened 117 infested mycelia and counted 23 potential transformants. Finally, 20 transformants were verified as positive, with a positivity rate of 17.09%.

### Expression levels of Hsp20 were up-regulated in transformants

**Figure 9:**
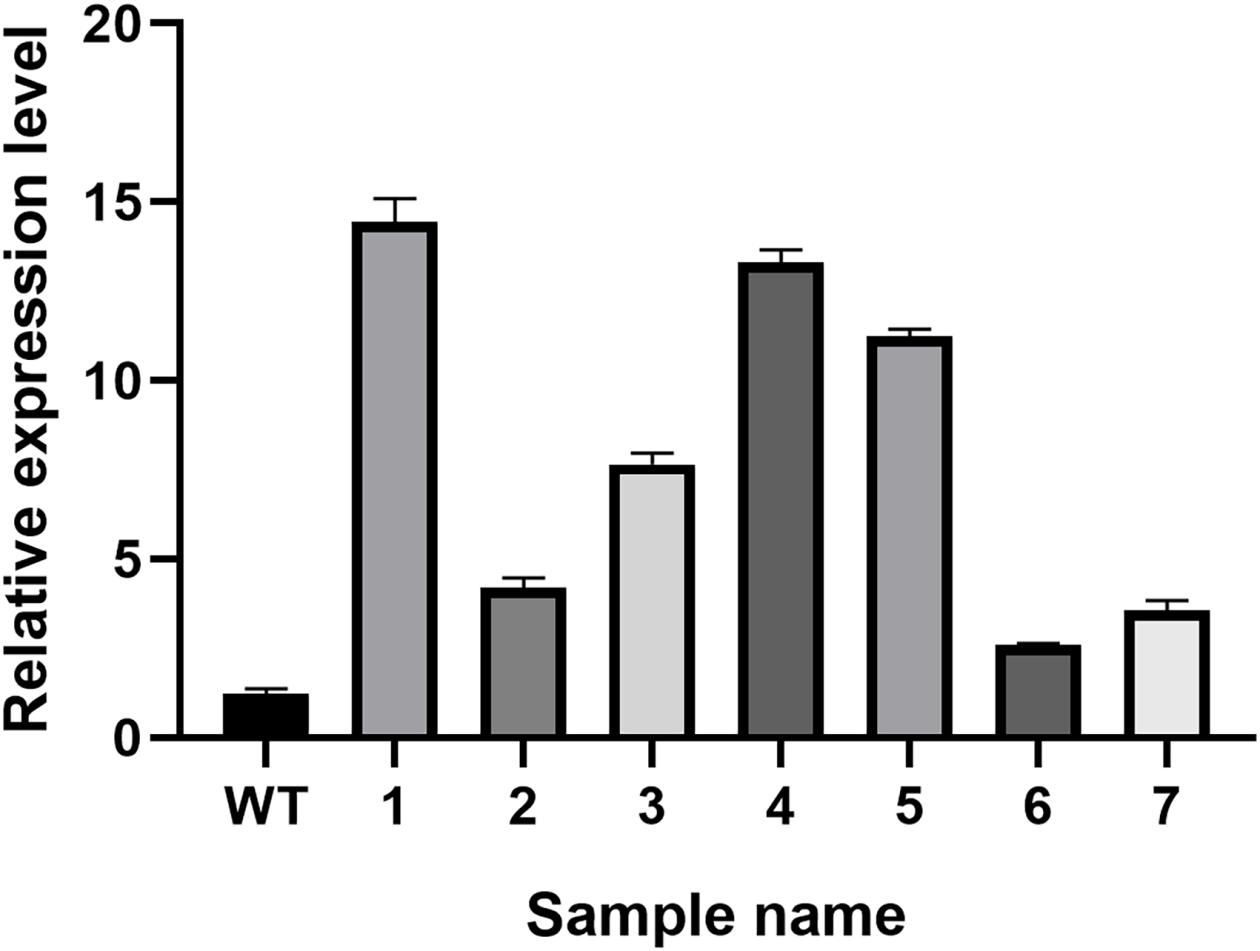
The qPCR analysis of transformants in *L. edodes.*

The real-time PCR experiment results of the seven randomly selected positive transformants (Figure 9) revealed the *hsp20* gene expression in the positive transformants was higher than in the control group, indicating that *hsp20* expression was overexpressed in *L. edodes*.

### Hsp20 overexpression enhances the mycelial growth of *Lentinus edodes*

**Figure 10:**
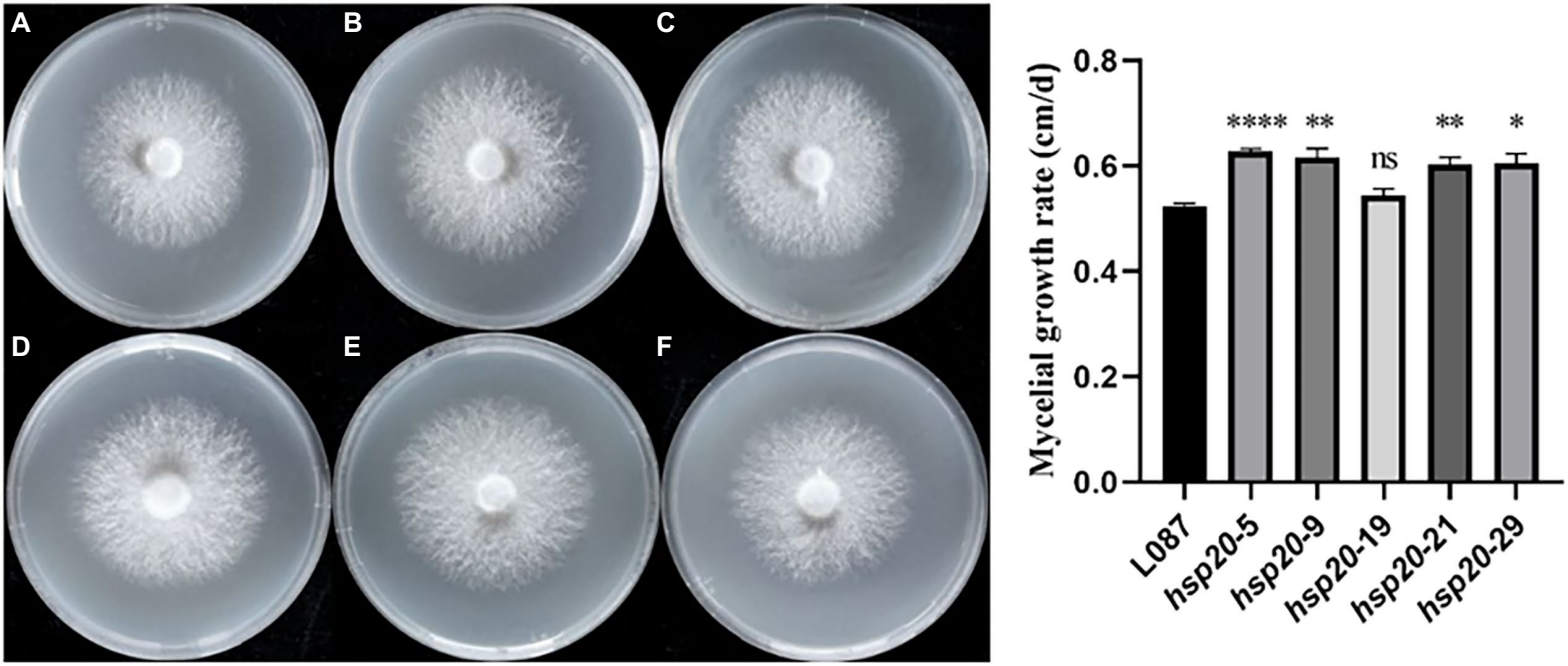
The growth of transformants on PDA media and growth rate. **(A)** L087, **(B)** hsp20-5, **(C)** hsp20-9, **(D)** hsp20-19, **(E)** hsp20-21, and **(F)** hsp20-29.

The colony morphology of the untransformed strain L087 and overexpressed transformants was examined, and the mycelial growth rate was determined. Figure 10 shows the growth of mycelia, with most transformants having larger colonies than the untransformed strain. In spite of this, the colonies did not differ significantly in terms of their morphology. The diameter of the colony and its growth rate were measured daily. The results of a one-way ANOVA (α = 0.05) are illustrated in Figure 10, with most transformants (except hsp20-19) showing statistically significant difference in mycelial growth compared to the untransformed control group. Transformants have faster mycelial growth than untransformed controls. The results of this study demonstrate that improving the expression of *hsp20* could promote the growth of *L. edodes* mycelia in PDA medium.

### Hsp20 overexpression improves the mycelial thermotolerance of *Lentinus edodes*

**Figure 11:**
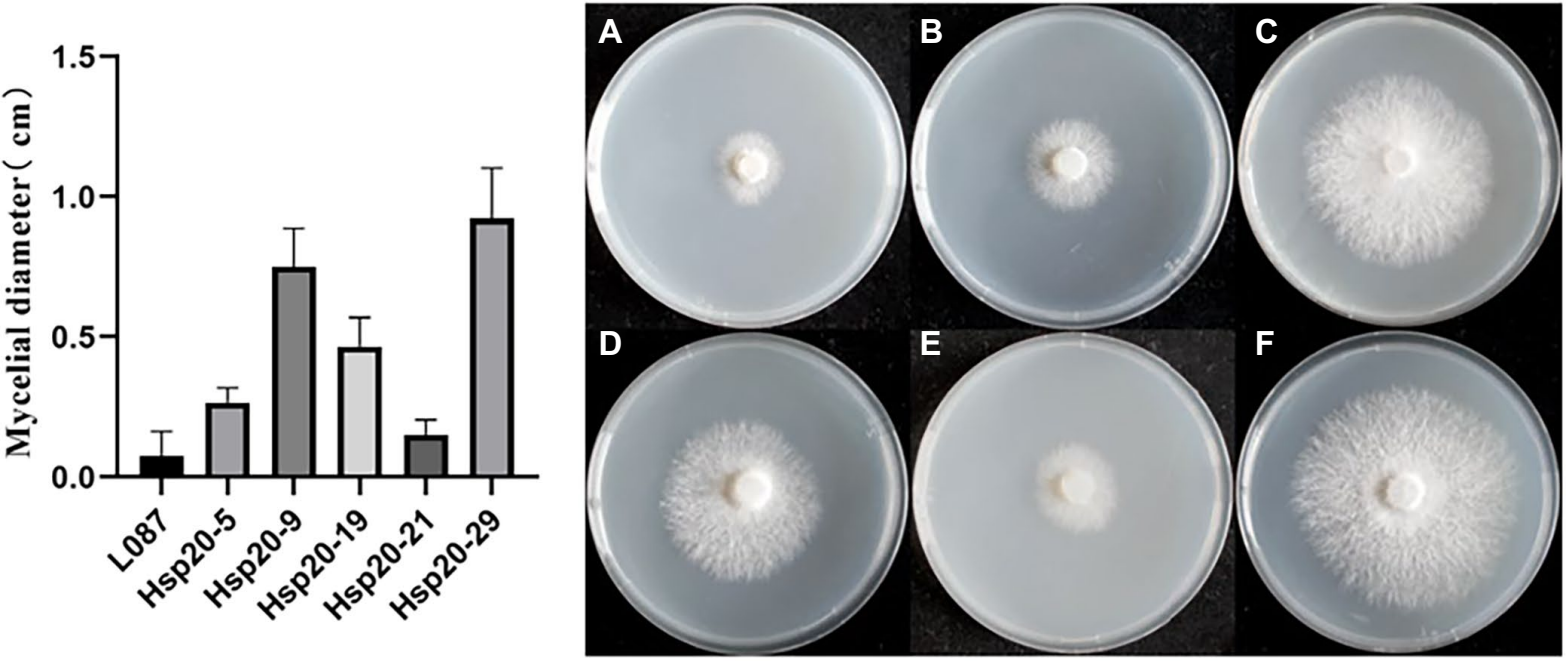
The growth of transformants after heat treatment. **(A)** L087, **(B)** hsp20-5, **(C)** hsp20-9, **(D)** hsp20-19, **(E)** hsp20-21, and **(F)** hsp20-29.

The difference in colony diameter before and after heat treatment was shown in Figure 11. These results showed that the transformants hsp20-29 had the best growth, with an average diameter increase was 0.925 cm compared to the pre-thermal treatment; hsp20-9 was second, with the average colonies diameter increase was 0.75 cm compared to before the heat treatment; hsp20-9 was third, with the average colonies diameter increase was 0.467 cm; While untransformed L087 were unable to recover growth. Figure 11 shows the mycelial growth of untransformed L087 and transformants on day 12 after heat treatment. It was observed that several transformants extended as usual, recovered their distinctive white color, had a cottony appearance and there was an obvious recovery of growth; while untransformed L087 could hardly recover its growth.

**Figure 12:**
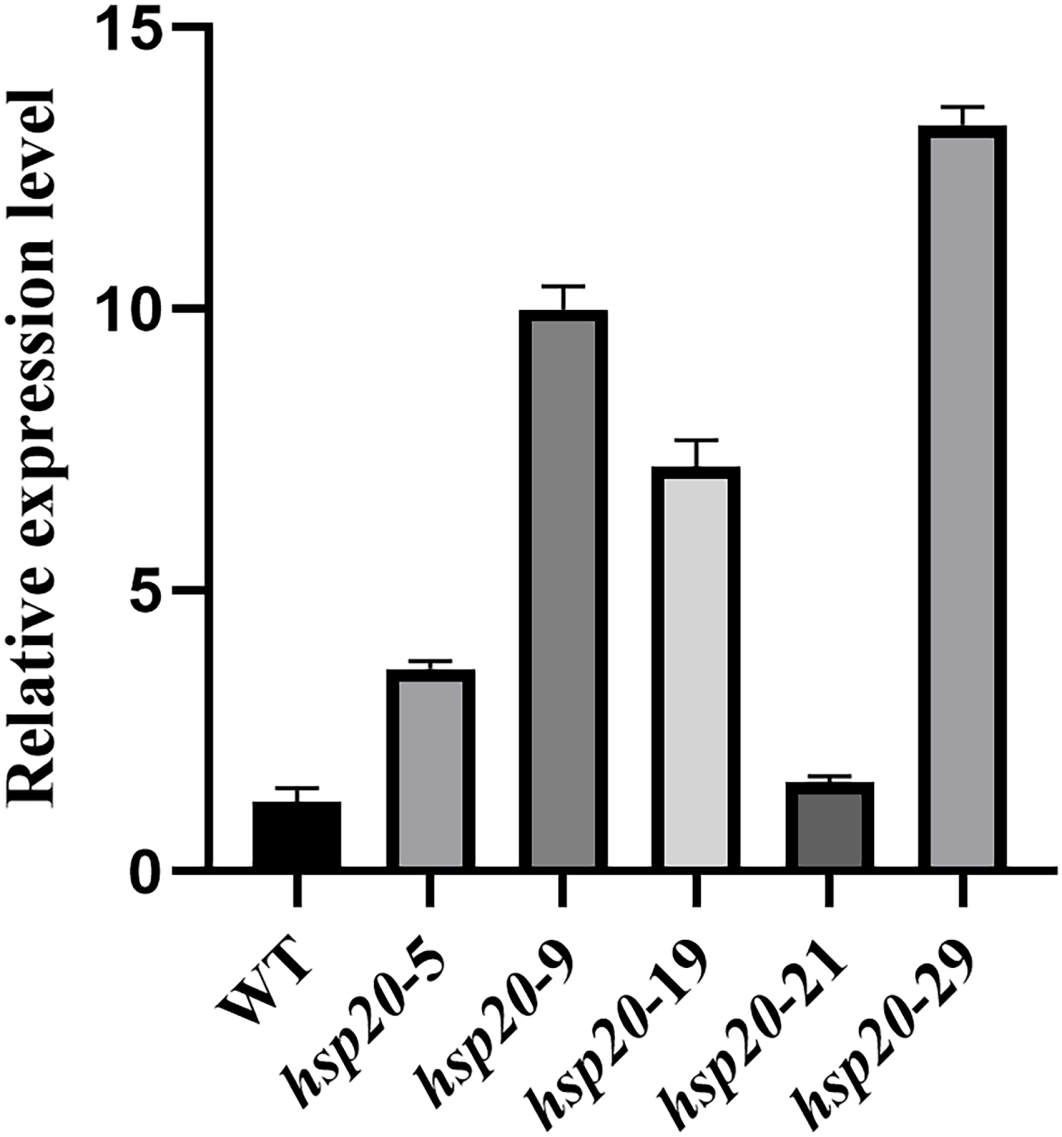
The qPCR analysis of transformants in *L. edodes.*

Transformants and untransformed L087 were examined for *hsp20* expression using real-time PCR, and the relative expression was calculated using the 2-Δct method (Figure 12). The results showed that transformants hsp20-29 had the highest gene expression, with hsp20 gene expression up-regulated more than 10-fold; untransformed L087 and the transformants hsp20-21 had the lowest gene expression. This work demonstrates that the transcription of *hsp20* for transformants was positively correlated with growth after heat treatment and that overexpression of Hsp20 may provide tolerance to high temperature for *L. edodes*.

## Discussion

*Lentinus edodes*, also known as shiitake, are cultivated and consumed throughout the world as a common edible and medicinal mushroom. It is common for high-temperature stress during culture to affect the growth of *L. edodes* mycelia and even causes mycelia apoptosis and rot, resulting in severe economic losses.

According to the literature currently, mycelia as an acceptor for transformation experiments can avoid the loss of transformants during the transmission process (Poyedinok and Blume, 2018). In this study, we used ATMT method to transform two cultivars of *L. edodes*, including dikaryotic and monokaryotic mycelia, each of which has its advantages. We achieved a total of 81 stably expressed transformants of *L. edodes.* Dikaryon germination frequency was 16.52%, and monokaryon germination frequency was 21.74%. In this study, monokaryotic mycelia had a higher transformation efficiency comparison to dikaryotic mycelia. The monokaryon was characterized by its denser mycelia, slow-growing, firm, neat form, and greater resistance to antibiotics. We speculate that the higher transformation efficiency of monokaryotic mycelia is related to their better growth ability. As a transformation material, monokaryotic mycelia has disadvantages as well. In the early stage of the study, we chose several monokaryon for hygromycin sensitivity experiments and discovered that the sensitivity levels varied widely amongst individuals, with some monokaryon still able to grow on MYG medium with hygromycin greater than 30 μg/ml. This concentration value is far higher than that typically used for transformation with hygromycin (Jiang et al., 2019). This result makes it difficult to define the hygromycin screening concentration for monokaryotic mycelia and can also lead to inaccurate experiments with falsely high transformation efficiency. In addition, monokaryotic mycelia typically grow slowly. Due to the extensive transfer screening required for transformation, the experiment time is greatly increased by the growth rate of monokaryon. Monokaryotic mycelia were more stable than dikaryon in transformation screening, which is consistent with previous studies (Li et al., 2019). The problem with dikaryotic mycelia as a transgenic acceptor is that the target gene may be diluted or even lost in the passages when the mycelia grow to the fruiting body stage of development, as reported in the study of *A. bisporus* (Poyedinok and Blume, 2018). We can solve this problem by preparing and regenerating protoplasts from heterokaryotic transformants. Considering the time saving and efficiency, dikaryotic mycelia as a transgenic acceptor is advisable based on the results of this study. We also found that the genotype of the transgenic acceptor can affect the efficiency of genetic transformation in fungi, in agreement with studies in plants (Hayta et al., 2021). In the early stage of the experiment, we selected three low-temperature dikaryotic strains as the transgenic acceptor, namely “Huanong-1,” “L087” and “Sengyuan-8404,” of which “Huanong-1” and “Senyuan-8404” were not screened for transformants, while “L087” was screened for stable transformants in each transformation. Based on the large influence of genetic background, it can be assumed that variety differences play a significant role in the variation in the efficiency of integration of T-DNA into the genomes (Yan et al., 2019). Therefore, choosing a suitable variety is crucial for genetic transformation or function investigation. Previous studies have shown that transformation efficiency can be improved by changing the medium used to culture the transformed receptor (Maleki et al., 2018). In the pre-experimental phase, we used PDA medium, MYG medium, and wood chip medium to culture mycelia, it grew best on the wood chip medium. However, the mycelia in the wood chip medium were more resistant to hygromycin and it is difficult to define its lethal concentration. It is important to note that nutrient-rich media would improve the tolerance of strain to hygromycin, and changing the screening medium would require redefining the hygromycin concentration to avoid false positives. This point is rarely mentioned in the literature. In addition, after co-cultivation of *A. tumefaciens* with mycelia needs to be incubated at 25°C for more than 1 week before the germination of the proposed transformants can be seen. The delayed integration of exogenous nucleic acids into the chromosomes would lead to slower growth of the initial transformants.

A category of proteins known as heat stress protein is generated by organisms in response to high temperatures. Studies have shown that heat stress proteins are widespread throughout the biological world, from bacteria to higher organisms, and that they play an important role in almost all living cells (Ghosh et al., 2018). Numerous studies have shown that the introduction of small molecule heat stress proteins genes-related into organisms can improve their heat resistance (Sanmiya et al., 2004; Wang et al., 2018b). In this study, we successfully expressed the *hsp20* gene in *L. edodes* mycelia. Comparing the original strain and the transformants, there was no significant difference between their mycelia and colony morphology, while the mycelial growth rate was significantly faster than the original strain. We hypothesized that increasing the expression of hsp20 could promote the growth of *L. edodes* mycelia in PDA medium. Meanwhile, we observed that both original strain and transformants showed the mycelia become thin, the color turned slightly yellow and dried out after 24 h of heat treatment at 40°C. However, some of the transformants recovered mycelial growth after being removed from the heat treatment and moved to incubation at 24°C, whereas the original strains did not. The difference in phenotypic traits between the transformants and control strains after heat stress indicates that *hsp20* overexpression can contribute to the recovery of mycelial growth after heat stress, further proving that *hsp20* gene can improve thermotolerance of low-temperature strains of *L. edodes*. In the present study, heat tolerance was only improved in the mycelial stage of *L. edodes*, but it remains to be examined whether the *hsp20* gene plays a function in the fruiting body stage and during growth.

## Data availability statement

The datasets presented in this study can be found in online repositories. The names of the repository/repositories and accession number(s) can be found in the article/Supplementary material.

## Author contributions

R-LZ was in charge of acquiring finance. Y-YL performed the experiment, analyzed the data, and wrote the manuscript. R-LZ and Z-LL assisted with the analysis through constructive conversations. All authors contributed to the article and approved the submitted version.

## Funding

This work was supported by the National Natural Science Foundation of China (Project ID: 31961143010, 31970010, and 31470152), Beijing Innovative Consortium of Agriculture Research System (Project ID: BAIC05-2022), CAS Engineering Laboratory for Advanced Microbial Technology of Agriculture KFJ-PTXM-016, and Henan Province Key Research and Development project “Precise breeding and directional development of important edible fungi germplasm” (Project ID: 221111110600).

## Conflict of interest

The authors declare that the research was conducted in the absence of any commercial or financial relationships that could be construed as a potential conflict of interest.

## Publisher’s note

All claims expressed in this article are solely those of the authors and do not necessarily represent those of their affiliated organizations, or those of the publisher, the editors and the reviewers. Any product that may be evaluated in this article, or claim that may be made by its manufacturer, is not guaranteed or endorsed by the publisher.

## Footnotes

^1^ https://www.ncbi.nlm.nih.gov/tools/primer-blast/
