# Supplementary material for: Construction of a heat-resistant strain of Lentinus edodes by fungal Hsp20 protein overexpression and genetic transformation
Source: Front Microbiol. 2022 Nov 17;13:1009885. doi: 10.3389/fmicb.2022.1009885 (PMC9721462; doi:10.3389/fmicb.2022.1009885)
Supplement: Supplementary file 1 [file Data_Sheet_1.docx]

Appendix 1. The primers used in this study.

| Primer | Sequence (5' to 3') |
| --- | --- |
| Legpd-F | **GGATCC**CGAAGTTTGAGGTGGTTGCG |
| Legpd-R | ATTCAAGCAGTCAATGGATT |
| Legpd-F1 | ATTCGAGCTCGGTACCCGG**GGATCC**CGAAGTTTGAGGTGGTTGCG |
| Legpd-R1 | CCTTTACTAGTCAGATCTA**CCATGG**ATTCAAGCAGTCAATGGATT |
| Legpd-F2 | GATCAGAAACTTCTCGACAGACGTCTAAGTTCGTCAGTTACCTAA |
| Legpd-R2 | **GAATTC**GTAATCATGGTCATAGCTGGCTTCAAACTCCACCAACGC |
| Legpd-F3 | GCGTATCACCGTTGGGTGA**AAGCTT**CGAAGTTTGAGGTGGTTGCG |
| Legpd-R3 | CCAGTGAAAAGTTCTTCTCCTTTACTATTCAAGCAGTCAATGGAT |
| *hsp*20-F | ATGTCTACTTTCTTCTACGA |
| *hsp*20-R | TCACCCAACGGTGATACGCC |
| *hsp*20-F1 | AATCCATTGACTGCTTGAAT**CCATGG**ATGTCTACTTTCTTCTACG |
| *hsp*20-R1 | TGAAAAGTTCTTCTCCTTT**ACTAGT**TCACCCAACGGTGATACGCC |
| *hsp*20-F2 | CGACATCGACCGCCTTATCA |
| *hsp*20-R2 | TGGAGTACTTCCCAGAGCGA |
| *hyg*-F | CTATTTCTTTGCCCTCGGAC |
| *hyg*-R | ATGAAAAAGCCTGAACTCAC |
| *hyg*-F1 | GCGTCTGCTGCTCCATACAA |
| *hyg*-R1 | GCGAAGAATCTCGTGCTTTC |
| q*gapdh* -F | TCTTAGGCTACACTGAGGACC |
| q*gapdh* -R | GAACATTGTGGCAGTTTCG |
| q*hsp*20-F | CCCTCGACCTTTCAAGCCAA |
| q*hsp*20-R | GAGTCTGCCATTATGGGCGT |

Restriction enzyme cutting site were shown in bold script
