# Supplementary material for: Construction of a heat-resistant strain of Lentinus edodes by fungal Hsp20 protein overexpression and genetic transformation
Source: Front Microbiol. 2022 Nov 17;13:1009885. doi: 10.3389/fmicb.2022.1009885 (PMC9721462; doi:10.3389/fmicb.2022.1009885)
Supplement: Supplementary file 2 [file Data_Sheet_3.docx]

Appendix 3. The sequence information of Legpd-2

ATTCAAGCAGTCAATGGATTGGAGTGTATTTAGAATCGAAGGTTGTGGAACAAGGAAAAGATGCGGACGAAAAACACAACTGATCCTTTTATAGATAATCGCGCTTCAGTCAAATCTTGATTGCGTGATGTTGTGACTTCGACAATAGCCCTAAAAGTCTAGACCTTAAAACACTTTAGTACCTCGGACCTGCGATTGGGTAGGATTTATTGGCCGAGTCCTTTGGGATGGACTGCCAATCAGCGAACGTTCTTCAGTTCTATCGCCCCATGACTAAGGTTGCCTGGAACCGTGCTCGAGCTTTGGATGGAAGTGCCTTTGAAAGGCCCTGCAAAGTCTTGCAAATGCTACAGCTTCCTCCCTGGCCTGGGTTTGTTGTTCTAGAACTACTCTCATTCTTATCTTCTGCATACAACCATTTCATGATATCCCAGGATACACCGGTTTCACCGTAGTAGCCTTATGGATGCAACTTTGACGCACTGACAATCTGACTGATATCGACCAATAAAAATAGATAAAACTCCCTGTTCGAGTCCTTTCCGTGATACATGCGGCTCTCCGACTGCACTCTAACGCCGATAGGATACGGGCCCGCTCAGATAACCAGCATAAAGGGATGCTCATGCAACTCTTTTCGGTTTGGCGTAAAACCTGAACTCTCGCATCTACGACAAAACGGATTTATCTGGTGCATATGCAGAGCAAGGGATTAGAGGCAAGGCCTGAAACGAAATACCTCTCAACCGATGTATAATCACTCCCGAGTTAACTGCTATACTCCAGCTAAGGCCATCCTGTACATTAGCGCGACCGGACCCGGCCTCTTCACGAGCTTTCTTGGTCATTGTTCACTAAGCTAGTACGACAAGAGTCGCATCTCACTCTCAGTCTGGAAAGAGAAAAGATTCCGCTCGACGAAATCATAAAAATTCAGTGCAACTGATGTCATGTGACCGGGGATATGCCCGATATGGCTTGTCACAAGATATATCAGTTATGCTGCAACTTATGAGCGTATTCGCAACCACCTCAAACTTCG
