# Supplementary figures and images for: Construction of a heat-resistant strain of Lentinus edodes by fungal Hsp20 protein overexpression and genetic transformation
Source: Front Microbiol. 2022 Nov 17;13:1009885. doi: 10.3389/fmicb.2022.1009885 (PMC9721462; doi:10.3389/fmicb.2022.1009885)

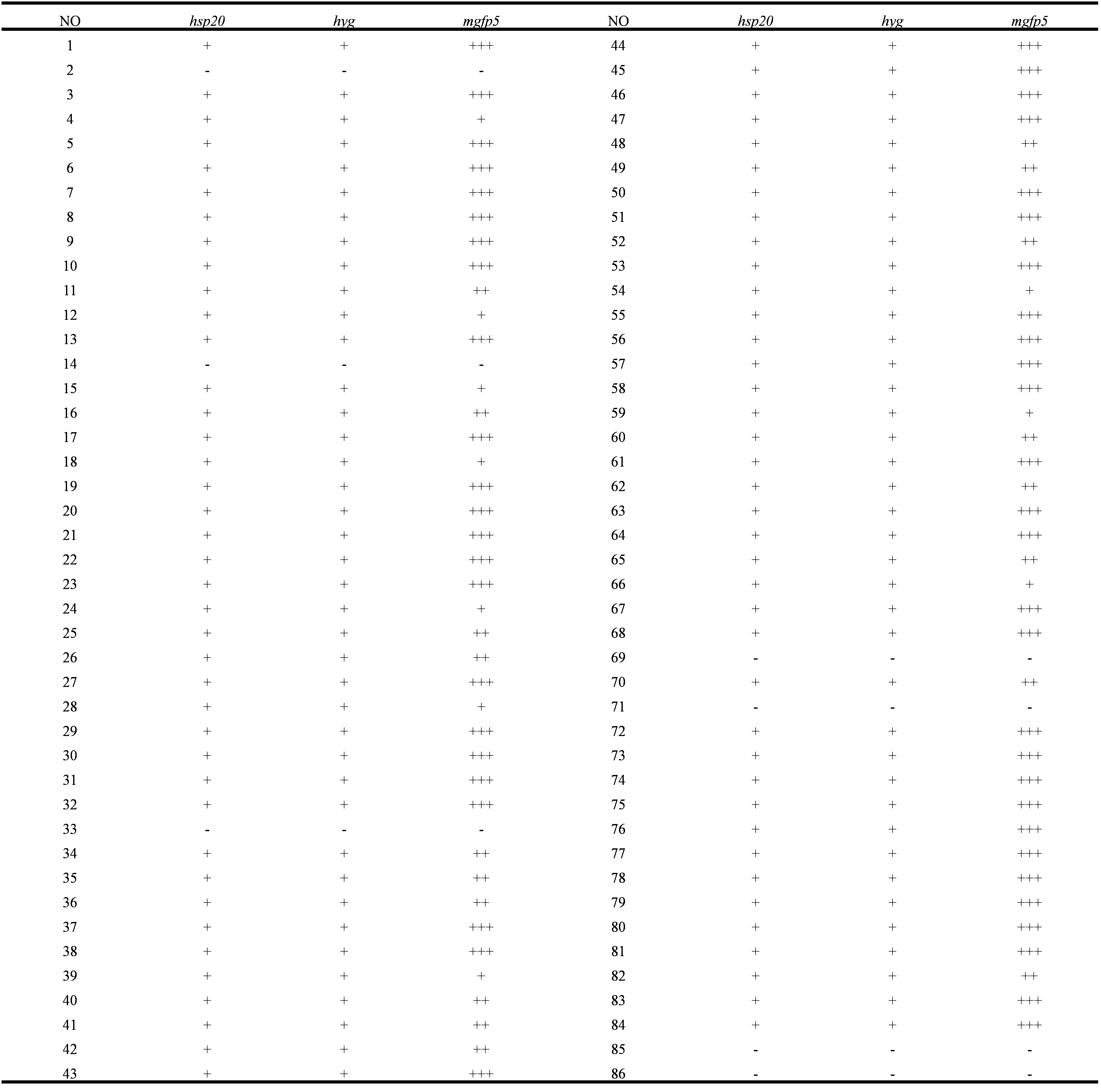

Supplement: Supplementary file 3 [file Image_1.TIF]
